# Comparative Study on the Quality of Microcrystalline and Epitaxial Silicon Films Produced by PECVD Using Identical SiF_4_ Based Process Conditions

**DOI:** 10.3390/ma14226947

**Published:** 2021-11-17

**Authors:** Mario Moreno, Arturo Ponce, Arturo Galindo, Eduardo Ortega, Alfredo Morales, Javier Flores, Roberto Ambrosio, Alfonso Torres, Luis Hernandez, Hector Vazquez-Leal, Gilles Patriarche, Pere Roca i Cabarrocas

**Affiliations:** 1 Electronics Department, Instituto Nacional de Astrofísica, Óptica y Electrónica, Puebla 72840, Mexico; arturo.ponce@utsa.edu (A.P.); alfredom@inaoep.mx (A.M.); atorres@inaoep.mx (A.T.); luish@inaoep.mx (L.H.); 2 Department of Physics and Astronomy, University of Texas at San Antonio, One UTSA Circle, San Antonio, TX 78249, USA; arturo.galindo@utsa.edu (A.G.); edoragui@gmail.com (E.O.); 3 Electronics Department, Benemérita Universidad Autónoma de Puebla, Puebla 72590, Mexico; javier.floresme@correo.buap.mx (J.F.); roberto.ambrosio@correo.buap.mx (R.A.); 4 Facultad de Instrumentación Electrónica, Universidad Veracruzana, Cto. Gonzalo Aguirre Beltran S/N, Xalapa 91000, Mexico; hvazquez@uv.mx; 5 Centre de Nanosciences et de Nanotechnologies (C2N), CNRS UMR 9001, Université Paris-Saclay, 911128 Palaiseau, France; gilles.patriarche@c2n.upsaclay.fr; 6 Laboratoire de Physique des Interfaces et des Couches Minces, CNRS, Ecole Polytechnique, Institut Polytechnique de Paris, 911128 Palaiseau, France; pere.roca@polytechnique.edu

**Keywords:** microcrystalline silicon, epitaxial growth, plasma enhanced chemical vapor deposition, electron microscopy

## Abstract

Hydrogenated microcrystalline silicon (µc-Si:H) and epitaxial silicon (epi-Si) films have been produced from SiF_4_, H_2_ and Ar mixtures by plasma enhanced chemical vapor deposition (PECVD) at 200 °C. Here, both films were produced using identical deposition conditions, to determine if the conditions for producing µc-Si with the largest crystalline fraction (X_C_), will also result in epi-Si films that encompass the best quality and largest crystalline silicon (c-Si) fraction. Both characteristics are of importance for the development of thin film transistors (TFTs), thin film solar cells and novel 3D devices since epi-Si films can be grown or etched in a selective manner. Therefore, we have distinguished that the H_2_/SiF_4_ ratio affects the X_C_ of µc-Si, the c-Si fraction in epi-Si films, and the structure of the epi-Si/c-Si interface. Raman and UV-Vis ellipsometry were used to evaluate the crystalline volume fraction (Xc) and composition of the deposited layers, while the structure of the films were inspected by high resolution transmission electron microscopy (HRTEM). Notably, the conditions for producing µc-Si with the largest X_C_ are different in comparison to the fabrication conditions of epi-Si films with the best quality and largest c-Si fraction.

## 1. Introduction

Microcrystalline silicon (μc-Si:H) has demonstrated to be an important material for the development and fabrication of large area semiconductor devices [1,2,3]. A widely used method to deposit these films is plasma enhanced chemical vapor deposition (PECVD) at low temperatures (200 °C) by employing a combination of SiH_4_ and H_2_ gas mixtures [4,5]. Currently, thin film solar cells and thin film transistors (TFTs) incorporate μc-Si:H films into their fabrication processes [6,7,8,9,10]. For tandem solar cells (μc-Si:H/a-Si:H), it is necessary to have large infrared absorption in the microcrystalline silicon film, which is achieved with a large crystalline fraction (X_C_). In like manner, a high electron mobility (µe) in the active layer is required to have faster devices with better performance characteristics, which is achieved with a μc-Si:H film with a large X_C_.

Microcrystalline silicon exhibits intermediate properties between hydrogenated amorphous silicon (a-Si:H) and polycrystalline silicon (poly-Si). In comparison with a-Si:H, μc-Si:H is more stable against light soaking and displays higher electron mobility (μ_e_) [11,12]. Alternatively, poly-Si has even better transport properties; however, it requires deposition temperatures of about 600 °C using low pressure chemical vapor deposition (LPCVD). The high temperature required for film deposition limits its integration capability on glass and flexible substrates. Previous studies have shown that µc-Si:H,F films produced by the dissociation of SiF_4_-H_2_-Ar and SiF_4_-H_2_-He gas mixtures by PECVD can yield a large crystalline fraction (X_C_) and large grain material [12,13,14,15].

On the other hand, epitaxial Silicon (epi-Si) films are receiving increased attention for the fabrication of novel devices, where c-Si films can be grown or etched in a selective way to produce 3D structures on the surface of the c-Si wafer, in addition to the production of ultrathin c-Si films [16,17,18] on foreign substrates [19], and epi-Si on metallic surfaces [20,21], crystalline semiconductors surfaces such as (100) c-Si [22] and gallium arsenide (c-GaAs) [23]. Moreover, the crystallization process using PECVD, which has been discussed in terms of the impact of silicon clusters [24] opens the possibility to produce novel devices at low substrate temperatures.

The deposition conditions for μc-Si:H and μc-Si:H,F resemble the conditions used to deposit epi-Si. While the transition from µc-Si:H to epitaxial growth has been reported in the case for the growth from SiH_4_-H_2_ gas mixtures, such study has not been carried out for µc-Si:H,F films obtained with the use of an SiF_4_ precursor, despite its interest in terms of the purity and properties of the films. In this work, we demonstrated the relationship between the deposition conditions and the Xc of µc-Si:H,F and epi-Si films. A systematic study was executed on μc-Si:H,F films produced from different SiF_4_, Ar and H_2_ mixtures, in order to understand the ongoing deposition mechanism and its effect on the silicon large grain (LG) formation. Likewise, the same deposition conditions were used to simultaneously study the quality of the epi-Si films and the (100) c-Si interface.

## 2. Materials and Methods

The µc-Si:H,F films were deposited on glass substrates (Corning, New York, NY, USA) by dissociation of SiF_4_, Ar and H_2_ gas mixture in a standard capacitively coupled PECVD reactor. A substrate temperature (T_s_) of 200 °C, reactor pressure (Press) of 2200 mTorr and radio frequency (RF) power of 25 W (corresponding to a RF power density of 260 mW/cm^2^) were employed to deposit the films. The H_2_/SiF_4_ gas ratio was varied in the range of 0.3–3.3 to determine its influence on the X_C_ of the films. The gas flow rates used were H_2_ = 1, 3, 5, 7 and 10 sccm, SiF_4_ = 3 sccm and Ar = 80 sccm. High argon dilution was used in combination with the SiF_4_ gas to promote the growth of nanocrystals in the film [13]. The epitaxial Si films were deposited on (100) c-Si wafers using the same conditions as for the µc-Si:H,F films deposited on glass.

The µc-Si:H,F films were characterized by Raman spectroscopy using an excitation energy wavelength of 633 nm. The crystalline volume fraction (X_C_) was calculated from the following relation X_C_ = (I_C_ + I_B_)/(I_C_ + I_B_ + I_A_), where I_C_, I_B_ and I_A_ are the integrated intensities of peaks associated to the crystalline phase at 520 cm^−1^, an intermediate phase that is correlated to small crystal sizes at 500–514 cm^−1^, and the amorphous phase presented at 480 cm^−1^, respectively, as reported in [25].

UV-Visible spectroscopic ellipsometry Horiba JobinYvon—MWR UVISEL (Horiba Ltd., Kioto, Japan), with an angle of incidence of 70°, was used to measure the imaginary part of the pseudo-dielectric function (Im[ε]) on the µc-Si:H,F and c-Si films and c-Si/epi-Si interface. The thickness of the films and their structural composition were determined by modeling the ellipsometry data using the Bruggemann effective medium approximation [26,27].

The films were modeled as a three-layer structure, by fitting the Im[ε] using the software Delta Psi included with the UV-Visible spectroscopic ellipsometer MWR UVISEL (Horiba Ltd., Kioto, Japan). The film is modeled as a three-layer structure consisting of: (1) a thin incubation layer, (2) a thick bulk layer and (3) a very thin surface layer (surface roughness). The composition of each layer can contain fractions of small silicon grains (SG), large silicon grains (LG), amorphous silicon (a-Si:H), voids and silicon oxide (SiO_2_).

Finally, the microstructural analysis of the µc-Si:H,F and epi-Si films and interfaces was performed by means of conventional transmission electron microscopy JEM 2200FS (Jeol Ltd., Tokyo, Japan) and high-resolution (HRTEM) ARM-200F (Jeol Ltd., Tokyo, Japan). The electron transparent samples were prepared in cross-section view using a focused ion beam (FIB), Zeiss Crossbeam 340, using a gradual process of 30, 10 and 2 kV energies of Ga^+^ ion irradiation. The TEM micrographs were recorded in a microscope ARM-200F (Jeol Ltd., Tokyo, Japan) microscope operated at 200 keV accelerating voltage.

## 3. Results

### 3.1. µc-Si:H,F Films

**Figure 1:**
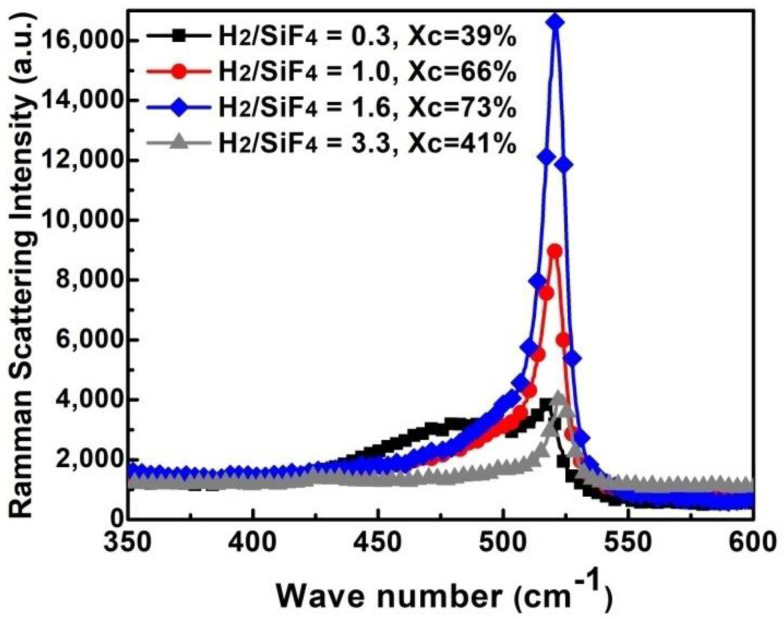
Raman spectra of µc-Si:H,F films deposited with a H_2_/SiF_4_ gas ratio of 0.3–3.3.

The crystalline fraction of the films (X_C_) was evaluated by using Raman scattering spectroscopy following the procedure described in the precedent section. The Raman spectra of the µc-Si:H,F films deposited with H_2_/SiF_4_ gas with ratios of 0.3, 1, 1.6 and 3.3 are shown in Figure 1. The spectra of the films deposited with a small H_2_/SiF_4_ gas ratio (H_2_/SiF_4_ = 0.3) resulted in the lowest X_C_ (39%). As the H_2_/SiF_4_ gas ratio increases, X_C_ increases as well. The film deposited with a H_2_/SiF_4_ gas ratio of 1.6, displayed the largest X_C_ (73%). A further increase of the H_2_/SiF_4_ gas ratio resulted in a reduction of X_C._ The observations deduced in this characterization allows us to define an optimal H_2_/SiF_4_ ratio (1.6) for growing µc-Si:H,F films with a large X_C_ value.

**Figure 2:**
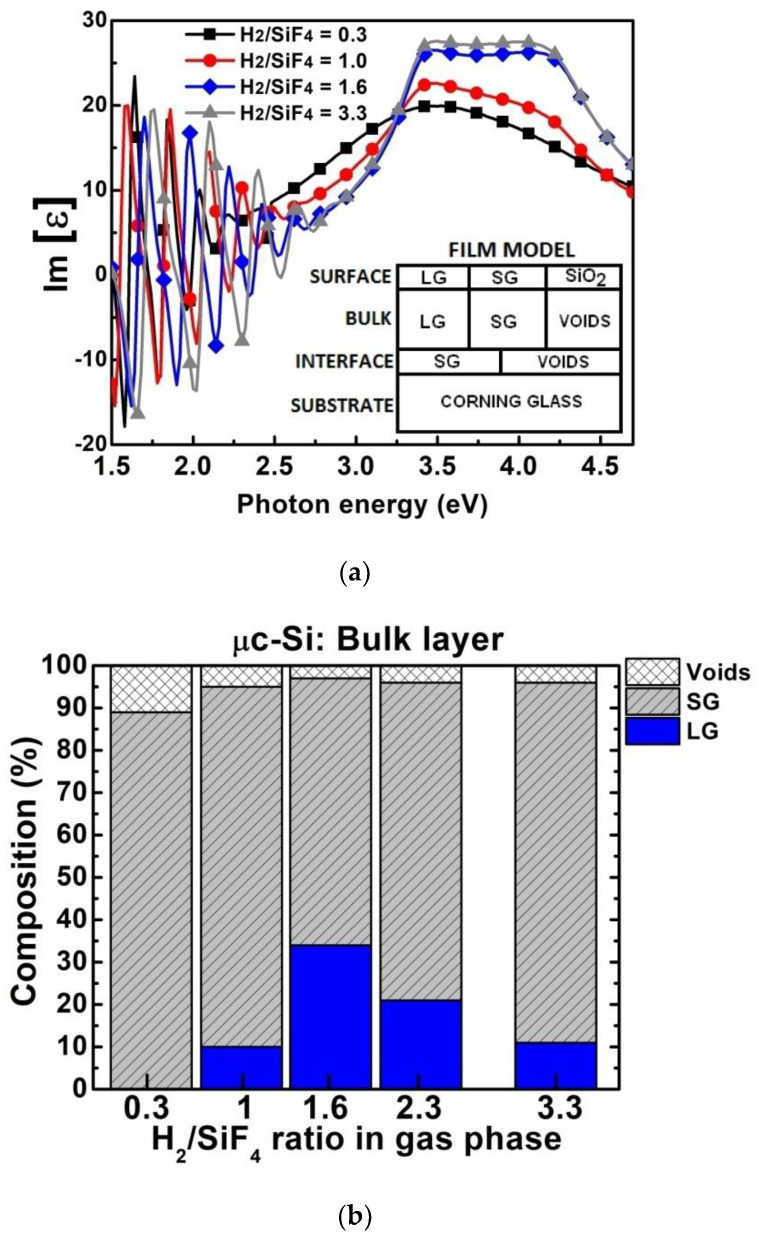
(**a**) Imaginary part of the pseudo-dielectric function (Im[ε]) of µc-Si:H,F films deposited with a H_2_/SiF_4_ gas ratio 0.3–3.3 on corning glass substrates. The inset shows the µc-Si:H,F film optical model. (**b**) Composition of µc-Si:H,F bulk films deposited on corning glass as a function of the H_2_/SiF_4_ gas ratio (obtained from modeling the Im[ε] spectra).

Raman spectroscopy was supported by performing spectroscopic ellipsometry on the same µc-Si:H,F films, where one measures the imaginary part of the pseudo-dielectric function (Im[ε]) versus the photon energy, as shown in Figure 2a. In this way, we employed the optical model based on the Bruggemann effective medium approximation (B-EMA) [26,27], where the modeled film is represented as a three-layer structure that consists of: (1)a thin incubation layer composed of small silicon grains (SG), amorphous silicon (a-Si:H) and voids; (2) a thick bulk layer composed of large silicon grains (LG), SG and voids; (3) a very thin surface layer (surface roughness) composed of LG, SG and silicon dioxide (SiO_2_). The dielectric functions for small grain and large grain silicon were taken from [26].

Figure 2b shows the structural composition (SG, LG and voids) of the µc-Si:H,F bulk layers as a function of the H_2_/SiF_4_ ratio. A H_2_/SiF_4_ ratio of 0.3 results in the growth of pure SG material (96%), the remaining 4% consisting of voids; however, when the H_2_/SiF_4_ ratio increases, the formation of LG appears in the film. By applying an H_2_/SiF_4_ ratio of 1.6, it results in a substantial LG fraction of 34%, along with SG and void fractions of 34% and 3%, respectively. A further increase of the H_2_/SiF_4_ ratio results in a decrease of the LG fraction. Notice that in all of the µc-Si:H,F films, the voids fraction is low, while the overall crystalline fraction (LG + SG) is large (~95%). No amorphous fraction could be detected in the bulk of all these samples.

Table 1 shows the thickness of each layer of the µc-Si:H films as a function of the H_2_/SiF_4_ ratio, as well the total film thickness and the deposition rate, while Table 2 shows the three-layer structure including the composition of each layer and the error of the model which lies in the range of 1.4–3%. The spectroscopic ellipsometry has higher sensitivity compared to Raman spectroscopy, enabling it to distinguish between LG, SG, amorphous, small cavities (voids) and SiO_2_ fractions.

In Table 2 also is observed that small and large H_2_/SiF_4_ ratios result in films with low silicon LG fraction, while an optimal ratio (H_2_/SiF_4_ = 1.6) results in µc-Si:H,F films with the largest silicon LG fraction. The largest X_C_ obtained using Raman spectroscopy in the bulk region was 73%, while the spectroscopic ellipsometry reported in the Table 2 shows an amount of 97% (LG = 34% + SG = 63%). Raman spectroscopy does not account for the presence of voids in the films, which can lead to a discrepancy with ellipsometry results as reported in [28].

**Figure 3:**
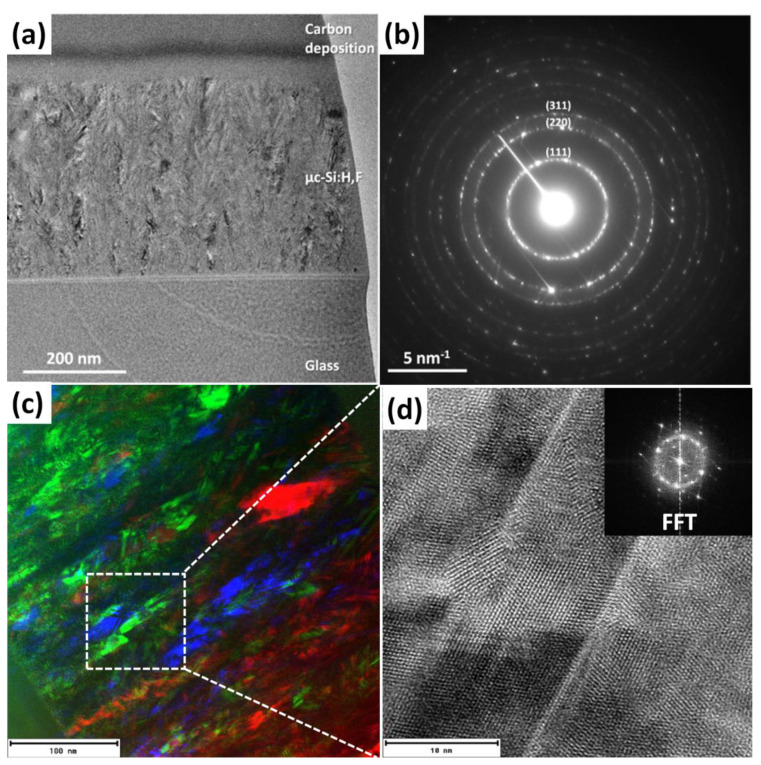
(**a**) TEM image of the µc-Si:H,F film, with the largest LG fraction (34%), and (**b**) SAED pattern of the film showing the characteristic planes of the Si structure. (**c**) RGB image formed by three dark field juxtaposed images collected with different positions of the objective lens aperture, and (**d**) HRTEM image of the selected area and their corresponding FFTs revealing the crystalline structure.

Figure 3a shows a cross section TEM image of the µc-Si:H,F film with the largest LG fraction. A selected area electron diffraction (SAED) pattern shown in Figure 3b indicates the formation of a microcrystalline film. The measured distances in the reciprocal space correspond to the (111), (220) and (311) family planes of silicon. HRTEM micrographs confirm the crystalline structure of the silicon film. Figure 3c shows three juxtaposed dark field images obtained with three different directions in the diffraction pattern of the µc-Si:H,F film, the RGB colors correspond to one of each of the positions where the objective lens aperture was located, producing an RGB multi-dark field crystallographic image. Figure 3d shows a magnified HRTEM image of a selected part of the film, where, observed,is the atomic order of various silicon grains, the inset is the fast Fourier transform (FFT) that indicate the crystalline nature of the growth film.

### 3.2. Epitaxial Silicon Films

The deposition of silicon films was also performed on (100) c-Si wafers by employing identical conditions to these used to grow µc-Si:H,F films on glass, as discussed in the previous subsection. However in this case, the native oxide on the c-Si surface was removed through a SiF_4_ plasma etching process for 5 min at a pressure of 30 mTorr and RF power of 0.1 W/cm^2^. Immediately after that, the same systematic procedure in terms of growing conditions was performed to study the microstructural behavior of the epi-Si films as a function of the H_2_/SiF_4_ gas ratio. As described on Section 3.1, we measured the pseudo-dielectric function (Im[ε]) of the silicon films deposited on (100) c-Si substrates by spectroscopic ellipsometry.

**Figure 4:**
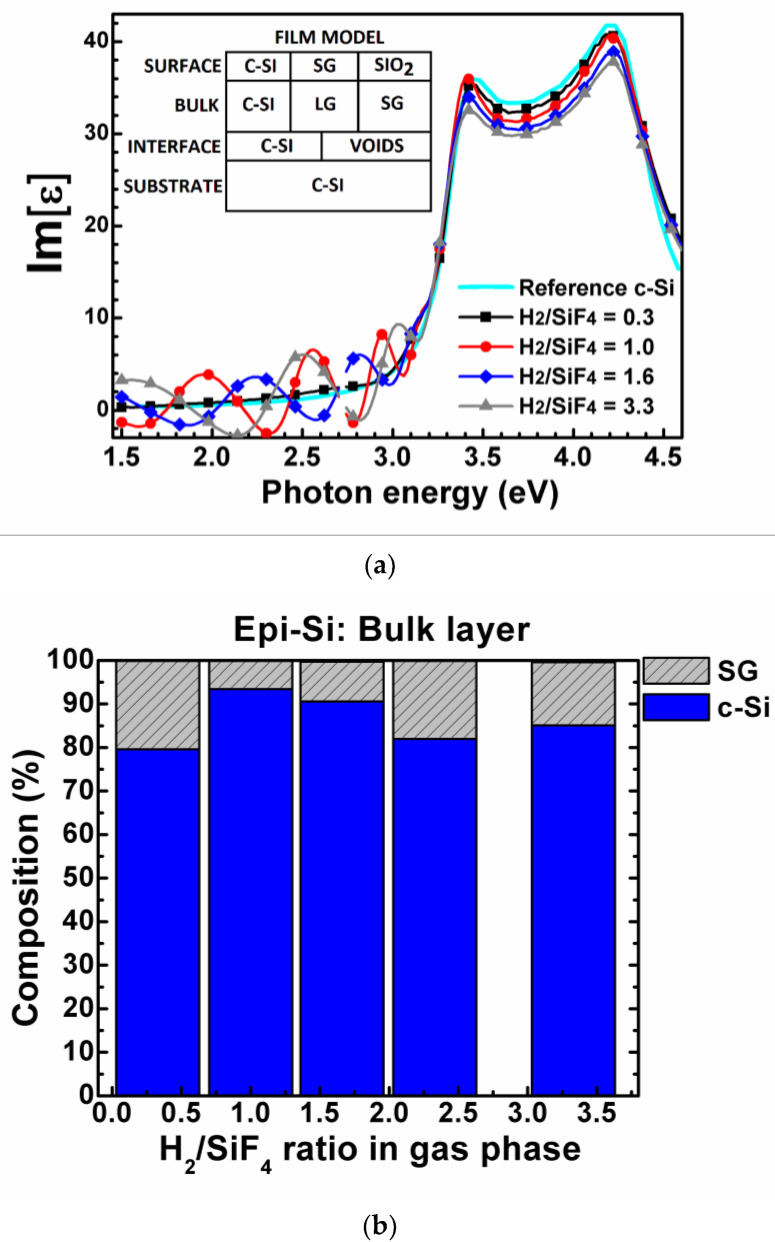
(**a**) Im[ε] spectra of epi-Si films deposited on bare (100) c-Si, with a H_2_/SiF_4_ gas ratio of 0.3–3.3. The inset shows the epi-Si film optical model. (**b**) Composition of the epi-Si bulk films deposited on c-Si, as a function of the H_2_/SiF_4_ gas ratio (obtained from modeling the Im[ε] spectra).

Figure 4a shows the Im[ε] spectra in the energy range of 1.5–4.6 eV. The films were deposited on (100) c-Si with a H_2_/SiF_4_ mixture in the range of 0.3–3.3. The film deposited with a H_2_/SiF_4_ ratio of 0.3 exhibited a Im[ε] spectra resembling that of (100) c-Si substrate, without interference fringes at low energy, thus indicating a perfect interface. However, when the H_2_/SiF_4_ ratio increases to 1, some interference fringes appear in the low energy range (1.5–3 eV). These fringes are related to the presence of voids (micro-cavities) at the c-Si/epi-Si film interface.

The inset in Figure 4a displays the optical model used to characterize the epi-Si films deposited on a (100) c-Si substrate. The epi-Si film is modeled as a three layer-structure consisting of: (1) a thin interface composed of c-Si and voids; (2) a thick bulk layer composed of c-Si, silicon LG and silicon SG; (3) a very thin surface layer (roughness) composed of silicon LG, silicon SG and silicon dioxide (SiO_2_).

Figure 4b shows the structural composition of the epi-Si bulk film (layer 2 of the model) as a function of the H_2_/SiF_4_ ratio. The c-Si fraction is large in all the epi-Si films (at least 80%). However, the film deposited with a H_2_/SiF_4_ ratio of 1 shows the largest c-Si fraction, which is close to 94%, the remaining 6% being SG silicon. Note that contrary to the µc-Si:H,F films on glass, no void fraction was detected in the epitaxial films.

**Figure 5:**
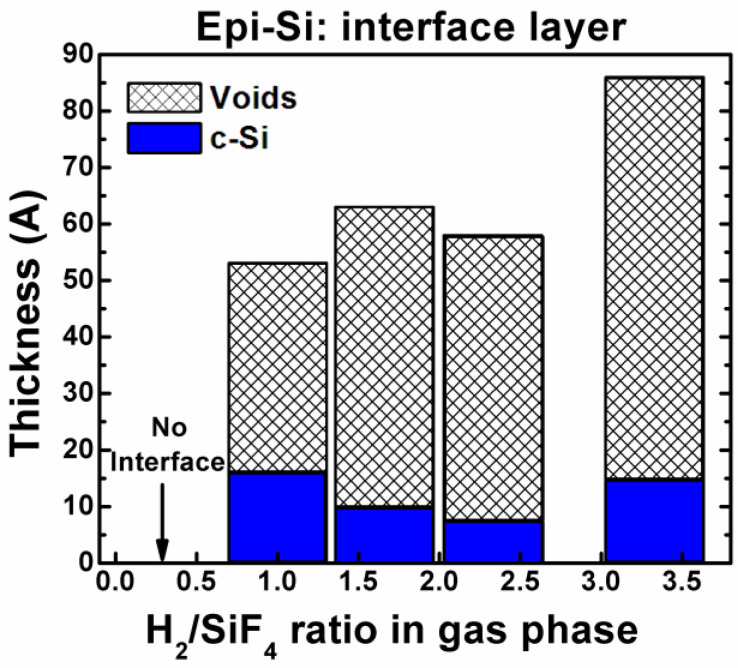
Thickness of the epi-Si interface layer with the c-Si substrate, as a function of the H_2_/SiF_4_ gas ratio.

In epi-Si films, the analysis of the epi-Si/c-Si interface is of utmost importance to determine the films capability to be integrated into various devices. Figure 5 shows the thickness of the epi-Si interface layer as a function of the H_2_/SiF_4_ ratio. Moreover, the compositional quantification of the epi-Si interfacial layer is shown. A small c-Si fraction and a large void fraction is observed (see Table 3). Table 3 shows the three-layer structure including the composition of each layer and the error of the model which lies in the range of 0.5–1.5%, while Table 4 shows the thickness of each layer of the epi-Si films as a function of the deposition H_2_/SiF_4_ ratio, as well the total film thickness and the deposition rate.

The epi-Si film deposited with a H_2_/SiF_4_ ratio of 0.3 does not exhibit an interfacial layer as seen in Table 3. The above indicates the absence of voids (nanocavities) at the epi-Si/c-Si interface. However, the interface layer appears in the epi-Si films deposited with larger H_2_/SiF_4_ ratios. Moreover, as the H_2_/SiF_4_ ratio increases, the generation of voids is promoted, while a thicker interface is produced.

**Figure 6:**
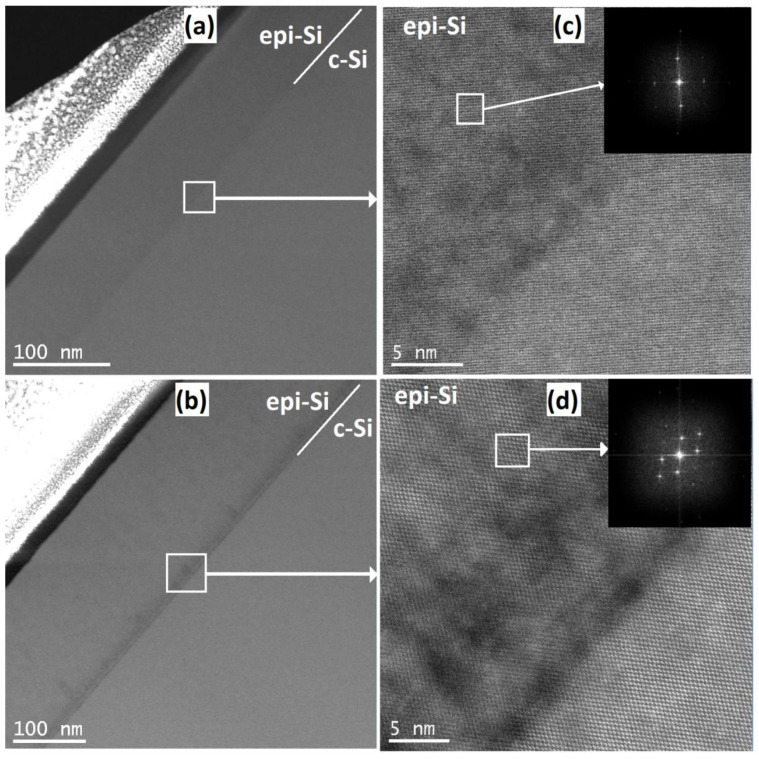
STEM cross sectional view of the epi-Si/silicon interface for the H_2_/SiF_4_ ratios of (**a**) 0.3 and (**b**) 1. HRSTEM on the black square region for the epi-Si films deposited using H_2_/SiF_4_ with mixing ratios of (**c**) 0.3 and (**d**) 1.

An in-depth assessment of the ep-Si/c-Si interface was performed using TEM analysis. Figure 6a,b shows two scanning transmission electron microscopy (STEM) images collected with the high angle annular dark field detector (STEM) of the epi-Si/c-Si interface deposited with a H_2_/SiF_4_ ratio of 0.3 and 1, respectively. While high resolution HAADF-STEM images are shown in the Figure 6c,d for the H_2_/SiF_4_ratio of 0.3 and 1, respectively.

The interface of the film deposited with a low gas ratio (H_2_/SiF_4_ = 0.3) is practically perfect (Figure 6c), and there is no evidence of large holes or nanocavities (black zones). On the other hand, the film deposited with a large gas ratio (H_2_/SiF_4_ = 1) has nanocavities at the interface (Figure 6d). Indeed, the variations of contrast in the high-angle annular dark-field STEM (HAADF-STEM) images are associated with changes of the material density between the c-Si film and the silicon substrate.

## 4. Discussion

**Figure 7:**
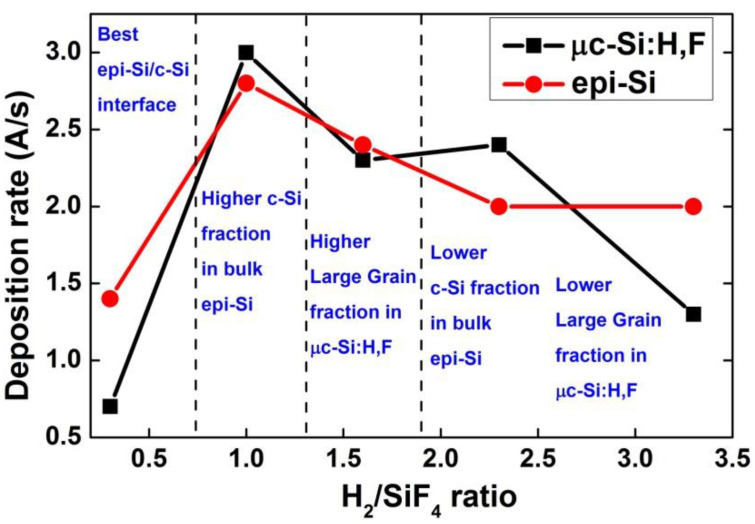
Deposition rate of µc-Si:H,F and epi-Si, as a function of the H_2_/SiF_4_ ratio.

In SiF_4_-H_2_-Ar plasmas, three regimes should be considered [29]: at low H_2_ flow rate, fluorine atoms are in excess and will etch any deposited material (low deposition rate); as the H_2_ flow rate is increased, HF will form in the plasma which can help to reduce the incorporation of oxygen in the films, so there is an optimum deposition rate and finally, when H_2_ is in excess, it will etch the deposited film, reducing the deposition rate. The above is shown in Figure 7, where is plotted the deposition rate of both films, as a function of the H_2_/SiF_4_ ratio.

For µc-Si:H,F deposition and epi-Si growth, a competition between the depositionand etching processes is determinant. For µc-Si:H,F there is an optimal H_2_/SiF_4_ ratio for producing the largest LG fraction (H_2_/SiF_4_ = 1.5). However, for epi-Si films, the H_2_ (H_2_/SiF_4_ ratio) is responsible for c-Si formation on the film and, also, for the formation of voids at the interface with the Si substrate.

In HRSTEM images, the voids (associated to a density deficit) in the c-Si/epi-Si interfaces are observed as black sections. In Figure 6d, it is clearly observed that the interface has a large amount of voids and nanocavities due to a large H_2_/SiF_4_ ratio, as opposed to Figure 6c, where the interface is smooth and practically free of voids corresponding to a film deposited with a low H_2_/SiF_4_ratio.

A low H_2_ flow rate (low H_2_/SiF_4_ ratio) is not enough to produce a high-quality epi-Si film, however, the outcome is an epi-Si film free of voids at the interfacial layer. Having a consistent flow rate between of both gases (H_2_/SiF_4_ = 1) creates larger c-Si fraction, while higher gas ratios result in lower epi-Si film quality with a thicker interfacial layer.

At last, the experimental outcomes discussed, highlight possible methods for use in the semiconductor industry. In the case of µc-Si it is very important to have large X_C_ for the development of devices. For example, thin film transistors (TFTs) require a high electron mobility (µ_e_) in the channel region to increase their performance. Therefore, a direct relationship exists between a large X_C_ and a large µ_e_ in µc-Si.

In like manner, thin film solar cells based on µc-Si also require a large X_C_. In our previous work [30], we demonstrated that higher X_C_ (and larger fraction of large grains) in the films provides a higher absorption on the infrared (IR) region of the electromagnetic spectrum, which is associated to a large short-circuit current density (J_SC_) and the efficiency of the solar cells.

In the case of epi-Si films, a perfect c-Si/epi-Si interface (low H_2_/SiF_4_ ratio) is of interest for the fabrication of novel 3D structures on c-Si wafers, by growing or etching the film in a selectively way. On the other hand, the growth of a defective interface (large H_2_/SiF_4_ ratio) can be advantageously used to influence the separation of the epi-Si film from the c-Si substrate; a process that cannot be achieved by conventional methods, as accomplished by H^+^ ion implantation in the smart cut process [31].

## 5. Conclusions

In this work, we have studied the effect of the H_2_/SiF_4_ ratio on the properties of silicon thin films deposited on glass and c-Si substrates. While the films on glass are microcrystalline silicon (µc-Si:H,F), epitaxial growth is obtained on (100) c-Si. The H_2_/SiF_4_ ratio used for the film deposition significantly affects the X_C_ in µc-Si:H,F, in addition to the quality of epi-Si and its interface with the silicon substrate. Three regimes have been identified: At low H_2_/SiF_4_ ratio, when F atoms are dominant in the plasma, the films are composed of small grain on glass and epi on c-Si, with a perfect interface. At intermediate values, the formation of HF in the plasma results in excellent µc-Si:H,F and epi-Si bulk properties, most likely related to the scavenging of oxygen impurities by HF on the growing film surface. At high H_2_/SiF_4_ ratios hydrogen is in excess and results in lower crystallinity for µc-Si:H,F and epi-Si films. Furthermore, we observed that the conditions used for producing µc-Si:H,F with the largest X_C_ are not exactly the same for producing epi-Si films with the best quality and largest c-Si fraction.

Large X_C_ in µc-Si:H,F is of much interest for the development of devices, as TFTs with a large µ_e_ (corresponding to films with large X_C_), which is required in the channel region to develop devices with better performance characteristics. For thin film solar cells based on µc-Si, high Xc provides a higher absorption of IR radiation, which is associated to a large J_SC_ and conversion efficiency.

For epi-Si films, a perfect c-Si/epi-Si interface is of interest for the fabrication of novel 3D structures on c-Si wafers, by growing or etching the film in a selectively way. On the other hand, the formation of a defective interface can be advantageously used to influence the separation of an epi-Si film from a c-Si substrate, analogous to the smart cut process.

## Figures and Tables

**Table 1:** Thickness of each layer of the µc-Si:H,F films deposited at different H_2_/SiF_4_ ratios, the total film thickness and deposition rate.

DepositionH_2_/SiF_4_ Ratio	Thickness (Å)				Deposition Rate(Å/s)
	Incubation Layer	Bulk Layer	Surface Layer	Total	
0.3	224	1823	58	2105	0.7
1	302	5148	49	5499	3
1.6	219	3899	48	4166	2.3
2.3	199	421	40	4450	2.4
3.3	286	3156	44	3486	1.3

**Table 2:** Structural composition of the µc-Si:H,F films deposited at different H_2_/SiF_4_ ratios according to ellipsometry measurements and models.

DepositionH_2_/SiF_4_ Ratio	Structural Composition			Model Error (%)
	Incubation Layer	Bulk Layer	Surface Layer	
0.3	SG = 0%	LG = 0%	LG = 0%	1.7
	a-Si:H = 98%	SG = 96%	SG = 63%	
	Voids = 2%	Voids = 4%	SiO_2_ = 37%	
1	SG = 0%	LG = 10%	LG = 16%	1.4
	a-Si:H = 81%	SG = 85%	SG = 46%	
	Voids = 19%	Voids = 5%	SiO_2_ = 38%	
1.6	SG = 0%	LG = 34%	LG = 29%	1.5
	a-Si:H = 94%	SG = 63%	SG = 63%	
	Voids = 6%	Voids = 3%	SiO_2_ = 33%	
2.3	SG = 0%	LG = 21%	LG = 27%	1.9
	a-Si:H = 100%	SG = 75%	SG = 41%	
	Voids = 0%	Voids = 4%	SiO_2_ = 32%	
3.3	SG = 94%	LG = 11%	LG = 12%	3
	a-Si:H = 6%	SG = 85%	SG = 58%	
	Voids = 0%	Voids = 4%	SiO_2_ = 30%	

**Table 3:** Structural composition of the epi-Si films deposited at different H_2_/SiF_4_ ratios according to ellipsometry measurements and models.

DepositionH_2_/SiF_4_ Ratio	Structural Composition			Model Error (%)
	Incubation Layer	Bulk Layer	Surface Layer	
0.3	No interface	SG = 20% c-Si = 80%	SG = 57% LG = 24% SiO_2_ = 19%	1.2
1	Voids = 69% c-Si = 31%	SG = 7% c-Si = 93%	SG = 28% LG = 54% SiO_2_ = 18%	0.5
1.6	Voids = 85% c-Si = 15%	SG = 9% c-Si = 91%	SG = 10% LG = 76% SiO_2_ = 14%	0.9
2.3	Voids = 87% c-Si = 13%	SG = 18% c-Si = 82%	SG = 19% LG = 62% SiO_2_ = 19%	1
3.3	Voids = 83% c-Si = 17%	SG = 15% c-Si = 85%	SG = 2% LG = 87% SiO_2_ = 11%	1.5

**Table 4:** Thickness of each layer of the epi-Si films deposited at different H_2_/SiF_4_ ratios, the total film thickness and deposition rate.

DepositionH_2_/SiF_4_ Ratio	Thickness (Å)				Deposition Rate (Å/s)
	Incubation Layer	Bulk Layer	Surfac Layer	Total	
0.3	No interface	877	9	886	1.4
1	53	1646	13	1712	2.8
1.6	63	1346	26	1435	2.4
2.3	57	1142	23	1222	2
3.3	86	1097	37	1220	2

## Data Availability

Not applicable.
